# A Frontier Review of Nutraceutical Chinese Yam

**DOI:** 10.3390/foods13101426

**Published:** 2024-05-07

**Authors:** Matthew Khol, Fanyi Ma, Lijing Lei, Wei Liu, Xiuhua Liu

**Affiliations:** 1 Henan International Joint Laboratory of Medicinal Plants Utilization, College of Chemistry and Molecular Sciences, Henan University, Zhengzhou 450046, China; 2 Henan Key Laboratory of Natural Medicine Innovation and Transformation, Henan University, Zhengzhou 450046, China; 3 School of Pharmacy, Henan University, Zhengzhou 450046, China; 4 State Key Laboratory of Antiviral Drugs, Henan University, Zhengzhou 450046, China

**Keywords:** *Dioscorea opposita* Thunb., Huai Shanyao, batatasin, polysaccharide, traditional Chinese medicine

## Abstract

Yams are the edible subterranean rhizomes, or tubers, of plants from the genus *Dioscorea*. There are approximately 600 species of yam plants in the world, with more than 90 of these growing in East Asia. One particular species, *Dioscorea opposita* Thunb., is highly praised as “the Chinese yam”. This distinction arises from millennia of storied history, both as a nutritional food source and as a principal ingredient in traditional Chinese medicine. Among the many cultivars of *Dioscorea opposita* Thunb., Huai Shanyao has been widely regarded as the best. This review surveyed the historical background, physiochemical composition, applications as food and medicine, and research prospects for the Chinese yam. Modern science is finally beginning to confirm the remarkable health benefits of this yam plant, long-known to the Chinese people. Chinese yam promises anti-diabetic, anti-oxidative, anti-inflammatory, immunomodulatory, anti-hyperlipidemic, anti-hypertensive, anti-cancer, and combination treatment applications, both as a functional food and as medicine.

## 1. Introduction

**Figure 1:**
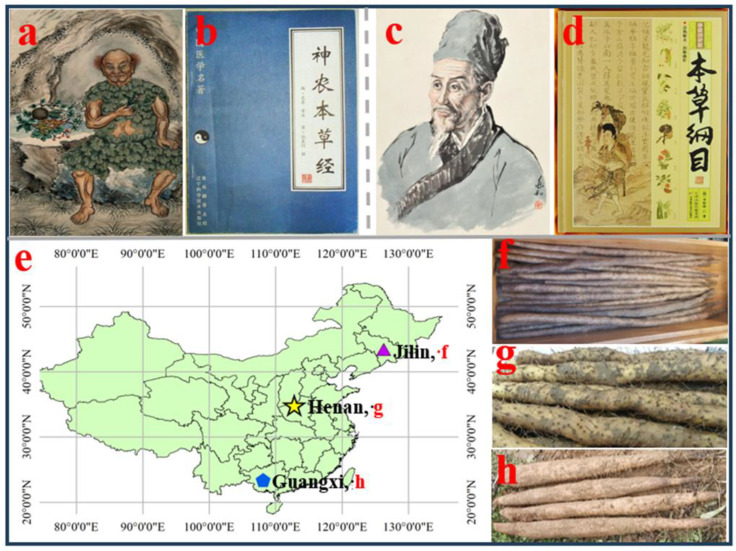
History and distribution of the Chinese yam in China. (**a**) Shen Nong “the Divine Farmer”; (**b**) Shen Nong Ben Cao Jing “Divine Farmer’s Classic of Materia Medica”; (**c**) Li Shizhen; (**d**) Ben Cao Gang Mu “Compendium of Materia Medica”; (**e**) Chinese yam cultivar distribution; (**f**) Xichangmao Shanyao in Jilin province; (**g**) Huai Shanyao in Henan province; (**h**) Jintian Huai Shanyao in Guangxi province.

Beginning with the oral tradition of the legendary Chinese figure Shen Nong (circa 2700 BC, Figure 1a), also known as “the Divine Farmer” [1], *Dioscorea opposita* Thunb. (*D. opposita*) first appeared in persistent text more than 2000 years later in the earliest Chinese pharmacopoeia, Shen Nong Ben Cao Jing (Figure 1b), which proudly proclaimed it “the Chinese yam” [2,3]. According to Li Shizhen (1518–1593 AD, Figure 1c), the author of Ben Cao Gang Mu (the Ming Dynasty pharmacopoeia, Figure 1d), the Chinese yam tuber was indicated as being effective in alleviating “indigestion, anorexia, diarrhea, and diabetes. It was suggested to have a hypoglycemic effect and promote the health of elderly women” [4].

Primarily prescribed as an invigorant in traditional Chinese medicine (TCM), Chinese yam has also been reported to improve coughing and dyspnea associated with lung deficiency [5], as well as to promote immune functions [6]. Yams are the edible underground rhizomes, or tubers, of the dioecious monocotyledon plant species of the genus *Dioscorea*, singular *Dioscoraceae*. They are a crucial staple food in the diets of many tropical and subtropical countries [7]. They are prized primarily for the carbohydrate nourishment they provide [3]. Globally, an estimated 88.3 million metric tons of yams were produced in 2022. The vast majority of these were cultivated in the “yam belt” of West Africa, with Nigeria, Ghana, and Cote d’Ivoire producing 61.2, 10.7, and 7.6 million metric tons, respectively [8]. There are an estimated 600 species of yam plants in the world, including more than 90 which grow in East Asia [9,10]. *D. opposita* has been a principal ingredient in the herbal medicine of China for millennia [11,12].

Today, the Chinese yam is a medicinal plant which is extensively cultivated in the People’s Republic of China (PRC). The harvest time is from late October until early December. The starchy tubers are typically blanched, or boiled quickly, to remove irritating oxalate crystals and residual pesticides from their skins, and then carefully cut into slices [13,14]. The next step is to dry them. The crude drug yam discs have been extensively used in “Chinese herbal medicine since ancient times to strengthen the functions of the spleen, kidney, liver, and stomach, to decrease phlegm, and heal fatigue, chronic diarrhea, and diabetes” [15].

In the PRC, Chinese yam is primarily grown and consumed in the Guangxi, Jiangsu, Henan, Shandong, Hebei, Shanxi, and Jilin provinces (Figure 1e). One particular cultivar of *D. opposita*, Huai Shanyao (HSY), has widely been considered to be the best, both as food and as medicine. HSY can be found in Jiaozuo city, in the Henan province (Figure 1g) [3,12,16]. Xichangmao Shanyao, in the Jilin province, is a cultivar of *D. opposita*, which was introduced and planted in northern China hundreds of years ago (Figure 1f). The Chinese yam also has another scientific name, *Dioscorea batatas* Decne., which is used in the southern parts of the PRC (Figure 1h) [17,18,19,20,21]. This name draws attention to the fact that the Chinese yam is the only species known to contain all five batatasins, which regulate the dormancy of temperate plants in the cold winter, instead of during the hot and dry season, as is the case with African yams [18,22].

Various *D. opposita* cultivars are available from the Wenxian Institute of Agricultural Sciences in Jiaozuo City, Henan Province. There is considerable diversity among different cultivars of Chinese yam. The HSY cultivar is “one of the four famous Huai Chinese traditional medicines with some special traits such as heavy tubers, oily quality, less tendon, sweet soothing, high medicinal effect, and boiling tolerance” [23]. This review surveyed the physiochemical composition, applications as food and medicine, and research prospects of *D. opposita*. A recent review by Li et al. summarized the nutritional and beneficial functions of Chinese yam [24]. Our current review, however, includes batatasins and excludes diogenin, which was not found by our research group specifically in the Chinese yam [25].

## 2. Chinese Yam Composition

The Chinese yam contains various chemical components, including starch, non-starch polysaccharides, fiber, protein, allantoin, dopamine, batatasins, phytic acid, choline, and ergosterol (Table 1). The Chinese yam is made up of approximately 13–27% carbohydrates, mostly starch [26]. According to a previous study, “In Chinese market, native starches are considered as health food because they are obtained from green botanical sources with undefiled properties” [27]. Yam tubers are also made up of 1–3% protein, but dried yam is more concentrated, comparable to cereal grains [28]. The major Chinese yam storage protein, comprising 90% of the total protein, is dioscorin [29]. Water makes up most of the remaining mass, approximately 67–84% [26].

Chinese yams hold both water-soluble fiber and insoluble fiber. Chemical studies of the Chinese yam have revealed other minor molecular constituents, such as abscisin II, allantoin, batatasins, choline, dopamine, glucoprotein, mannan, phytic acid, polysaccharides, and minerals such as calcium, copper, iron, magnesium, manganese, phosphorous, potassium, and zinc [10].

### 2.1. Starch

The Chinese yam starch type is the C-type, having both crystalline A-type and amorphous B-type [5]. Comparatively, potatoes are B-type. Among species of *Dioscorea*, starch crystallinity decreases with increased amylose content, while starch swelling power decreases with increased amylopectin chain-length, which also increases starch crystallinity. *D. opposita* has a high amylose to amylopectin ratio and is thus very viscous [30].

Starches undigested after 120 min postprandial are considered to be resistant starches [31]. Boiling Chinese yams can reduce their resistant starch content by 80% [32]. Resistant starches benefit short-chain fatty acid production in the large intestine, inhibiting hepatic cholesterol synthesis and suppressing colon cancer cell proliferation in vitro. “These effects are assumed to prevent the development of coronary heart disease and colon cancer” [32].

### 2.2. Polysaccharide

Chinese yam mucopolysaccharide (CYMP), or root-tip yam sugar, is composed of approximately 40% polysaccharide and 2% protein [33]. The polysaccharide fraction of CYMP contains mannose, fructose, galactose, xylose, and glucose [34]. The glucose and protein composition of Chinese yam tuber mucilage (CYTM) in dry weight was found to be 11.05% and 13.39%, respectively [35]. CYTM is largely wasted in industrial processes, but it shows tremendous promise as a natural food emulsifier and stabilizer [36,37,38].

Chinese yam polysaccharide (CYP) is typically separated from dried Chinese yam discs through extraction, and glucose and proteins have been found to comprise 63.25% and 0.21% of CYP, respectively [39]. CYP is a kind of carbohydrate polymer, primarily composed of mannose, glucose, galactose, and glucuronic acid [40]. CYP is regarded as an important bioactive natural product [41]. Once purified, CYP can be useful for several purposes, including anti-diabetic, anti-oxidative, anti-bacterial, immunomodulatory, and anti-cancer applications [42,43].

### 2.3. Protein

The widespread practice of blanching yams, before cutting them into discs and then drying them, reduces protein content. Drying yam discs at a relatively lower temperature preserves much more of the protein [44].

Found in Chinese yam tubers, but not in the leaves, dioscorins taken from different sources often have different structures and activities [45], but recombinant dioscorin conformational structures are similar to their corresponding native dioscorins [46]. Differences “were closely related to the loss of the glycosylation from the protein” [47]. Dioscorin exhibits carbonic anhydrase activity and weak trypsin inhibitor activity, which can slow protein digestion [48,49]. Dioscorin also shows monodehydroascorbate reductase activity and dehydroascorbate reductase activity [50]. Dioscorin even displays in vitro angiotensin converting enzyme inhibitory activity, and in vivo anti-hypertensive activity [51]. In vitro, dioscorin is a dose- and pH-dependent antioxidant against 1,1-diphenyl-2-picrylhydrazyl, and can also capture hydroxyl free radicals [29].

Other Chinese yam proteins include lectins, a group of proteins or glycoproteins that has carbohydrate binding capability. Chinese yams produce lectins of varying sizes: mannose-binding lectin (DB1) and two maltose-binding lectins [52]. DB1 is insecticidal and helps to preserve these yam tubers against pests [53].

### 2.4. Batatasins

**Figure 2:**
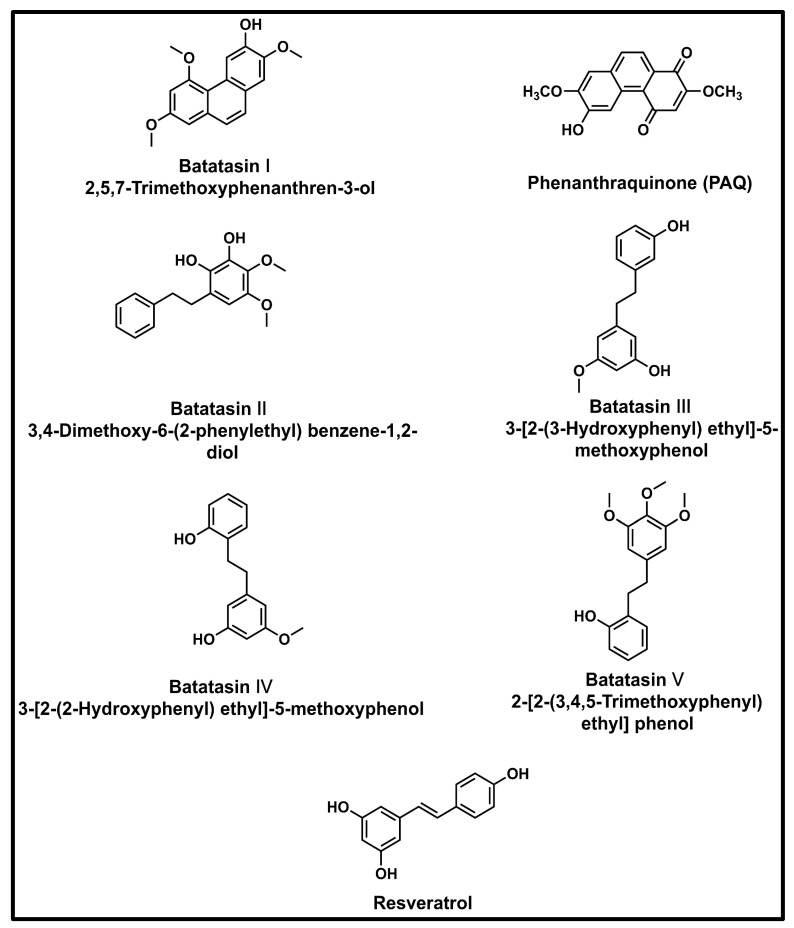
Batatasins I–V and analogues.

The primary phenolic compounds found in Chinese yams are known as batatasins. Originally, only dormant *D. opposita* aerial bulbils were thought to contain all five batatasins (Figure 2) [18]. While all five batatasins have not been found in any other species, they have now all been identified within Chinese yam rhizomes [54]. Batatasin I and another structural analogue, 6-hydroxy-2,7-dimethoxy-1,4-phenanthraquinone (PAQ), have been identified in Chinese yam rhizome methanol extract. These compounds are potential therapeutic treatments for allergic-inflammatory conditions [45,55].

The bibenzyl stilbene molecular structures of batatasins II–V are molecular analogues of resveratrol (RV), which is both a powerful natural antioxidant and inhibitor of cyclooxygenase-2 (COX-2) expression. The primary source of RV is grapes, *Vitis vinifera* [56]. With diverse therapeutic properties, RV has been well-studied as a nutritional supplement, and promises anti-diabetic, anti-oxidative, anti-inflammatory, immunomodulatory, cardio-protective, anti-cancer, and neuro-protective activities [57]. RV derivatives were explored as an adjunct therapy for elderly patients during the COVID-19 epidemic and were thought to offer anti-aging, anti-inflammatory, anti-oxidative, and anti-viral effects [58].

### 2.5. Other Small Molecular Constituents

**Figure 3:**
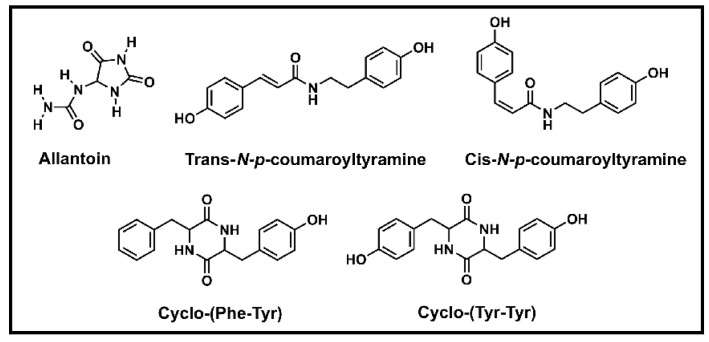
Allantoin, trans-*N-p*-coumaroyl tyramine, cis-*N-p*-coumaroyl tyramine, cyclo-(Phe-Tyr), and cyclo-(Tyr-Tyr).

The Chinese yam has a variety of small molecular constituents, such as allantoin, trans-*N-p*-coumaroyl tyramine (TCT), and the cyclic dipeptides etc. (Figure 3). Allantoin is more concentrated in Chinese yam skin, but is also found in the rhizomes. Allantoin promotes cell proliferation, speeds up healing, and soothes inflammation both internally and externally [34]. Allantoin is an easily synthesized small molecule and has long been used in medicinal and cosmetic preparations [59,60].

TCT was extracted from the HSY cultivar by our research group and had considerable inhibitory activity against α-glucosidase; TCT transforms into its cis-isomer, cis-*N-p*-coumaroyl tyramine, with no inhibition of α-glucosidase activity, under ultra-violet light [61]. The concentration of TCT is higher in the yam skin than inside the tuber [62].

Several other plants also contain TCT. For example, TCT from the hemp seeds of *Cannabis sativa* L. has been shown to exhibit melanogenesis and anti-tyrosinase activity [63,64], while TCT from the stems of *Dracaena usambarensis* Engl. has been shown to exhibit anti-inflammatory activity [65]. TCT α-glucosidase inhibitory activity, IC_50_, was 0.42 μM in *Tribulus terrestris* Linn. and 0.6 μM in *Huberantha jenkinsii* [66]. One study showed that TCT extract from *Tribulus terrestris* induced extrinsic, as well as intrinsic, apoptosis pathways in cancer cells [67].

Our research group has also found and isolated the cyclic dipeptides, cyclo-(Phe-Tyr) and cyclo-(Tyr-Tyr), from Chinese yam tubers [68]. Cyclic dipeptides are believed to have multiple important biological functions, such as anti-fungal, anti-bacterial, anti-cancer, immunomodulatory, anti-inflammatory, and anti-viral activities [69].

## 3. Chinese Yam as Food and Medicine

**Figure 4:**
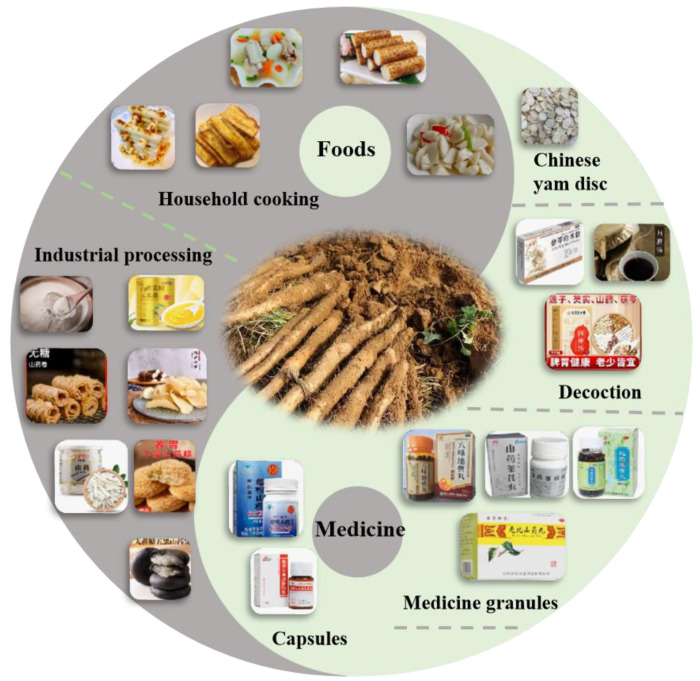
Chinese yam as food and medicine. Food: Household cooking and industrial processing. Medicine: Chinese yam discs, decoctions, medicinal granules, and capsules.

Chinese yam can be served as a nutritious and functional food, and it has several health benefits described in TCM (Figure 4):

… a sweet soothing herb that stimulates the stomach and spleen and has a tonic effect on the lungs and kidneys … The tuber is also anthelmintic and digestive. It is used internally in the treatment of poor appetite, chronic diarrhea, asthma, dry coughs, frequent or uncontrollable urination, diabetes, and emotional instability. It is applied externally to ulcers, boils, and abscesses.

[34]

### 3.1. Chinese Yam as Food

Chinese yam tubers can be stored for up to one year after harvesting and may be eaten without cooking. Globally, food yams are usually stored as whole tubers or processed into flour [17]. Freeze-drying yam flour preserves more antioxidant activity than hot air-drying or drum-drying [70,71].

The benefits of wheat flour, yam flour, and boxthorn yam noodles have also been explored, as functional food consumption continues to increase every year. BALB/c mice and humans share a more than 55% homology in the apoA-II amino acid sequence, showing lipid metabolism similarities. Experimental mice fed a yam-boxthorn noodle diet, rather than a strictly wheat flour noodle diet, had significantly lower total cholesterol and triglyceride serum levels [72]. The functional yam-boxthorn noodle could become a staple with hypolipidemic properties. Likely active lipid-lowering Chinese yam components include dietary fiber, mucilage, plant sterols, or a synergism of all these [10].

The addition of 0.2–0.6% weight/volume of *D. opposita* to yogurt can provide allantoin supplementation without making any modifications to the fermentation and storage process, highlighting one of potential applications of Chinese yam in fermented foods [73]. Microbial hydrolysis by fermentation enhances antioxidant activity in plant-based foods through increased release of phenolic compounds and flavonoids by breaking down cell walls [74]. Raw Chinese yam fermented by *Lactobacillus acidophilus* has been found to be a superior functional food, healing and preventing gastric lesions in rats [75].

The Japanese cultivar of *D. opposita* is known as “*tsukuneimo*” or “*iseimo*”. *Tsukuneimo tororo* tuber mucilage hydrolysates can be prepared with the digestive enzymes pepsin, trypsin, and papain. Enzymes are extremely useful tools for improving functional foods [76]. *Tsukuneimo tororo* soup “should be recognized as one of the health foods to prevent or improve lifestyle-related diseases such as cancer, diabetes, and hypertension” [77].

Yam peel portions are significantly better than the rhizome portions at scavenging reactive oxygen species (ROS) [78]. Recently, yam chips have become highly marketable in China. Yam chip preparation involves a heat pump and far-infrared radiation to achieve “the desired moisture ratio, the smallest shrinkage of dried Chinese yam chips, the highest rehydration percentage, and the smallest changes of color” [79].

CYTM has shown antioxidant activity in vitro [80]. CYTM is usually extracted with solvents, centrifugation, and heat, as it can be difficult to separate CYP and starch from CYTM. Bubble separation has recently been used as an innovative method to purify CYTM, as the process protects antioxidants from chemical damage and heat [81]. Once CYP and starch have been separated from CYTM, they may also serve as potent ingredients. When eaten, CYP promises immunomodulatory benefits [34]. Meanwhile, eating modified resistant yam starch was shown to prevent constipation and significantly improve serum lipid profiles in a murine model [82].

Dioscorin, as the major storage protein in Chinese yam and an antioxidant, may supplement health when eaten as a food additive or consumed in yam tubers [29,83]. It has been shown that consuming dioscorin-containing yam products reduces blood pressure in humans and stimulates the innate immune systems of mice [45].

Eating Chinese yams could also improve several disease conditions by reducing ROS. A Chinese yam-supplemented diet reduced azoxymethane-induced colon carcinogenesis among F344 rats by scavenging ROS, as well as through increased colonic mucosal gene expression of superoxide dismutase, and suppressed colonic mucosal gene expression of inflammatory mediators [84]. Continued Chinese yam food research will facilitate the production of processed foods with more antioxidant and anti-hypertensive activity [85]. Chinese yam has beneficial properties as food, and it also has medicinal benefits, such as anti-oxidative action, serum glucose reduction, and immune improvement [3]. Therefore, it is considered to be a nutraceutical (Table 2).

### 3.2. Chinese Yam as Medicine

Medicinal Chinese yam is harvested in the winter and processed by washing, peeling, drying, and slicing [10]. There are many pharmacological purposes for medicinal Chinese yam (Table 3).

#### 3.2.1. Anti-Diabetic

The high amylose to amylopectin ratio in Chinese yam slows digestion, a beneficial anti-diabetic activity. High amylose consumption, as opposed to high amylopectin consumption, significantly lowers serum glucose levels at 30 min and raises levels at 180 min, while lowering postprandial insulin levels at 30 min and 60 min [99].

Streptozotocin-injected rats mimic pre-diabetic status, and injections of Chinese yam extract every day has been shown to contribute to reduced fasting serum glucose levels in these rats [100], leading to improved kidney function [101]. In this same model, allantoin was shown to improve insulin resistance. Allantoin administration increased β-endorphin secretion from the adrenal glands of these rats, activating opioid μ-receptors to increase glucose transporter four expression, which increased skeletal muscle glucose uptake, thereby reducing the serum glucose level [86]. Another study confirmed that allantoin activates imidazoline I3 receptors and enhances insulin secretion, lowering serum glucose levels in the experimental rats [87].

Batatasin I and three other bibenzyls, isolated from the HSY tuber crude ethanol extract, have shown considerable α-glucosidase inhibitory activity. These α-glucosidase inhibitory compounds show promise as a potential new class of anti-diabetic drugs [61]. TCT, which our research group has isolated from HSY tubers, also shows considerable inhibitory activity against α-glucosidase (IC_50_ = 0.40 μM). As a carbohydrate-hydrolase, α-glucosidase releases glucose. Several α-glucosidase inhibitors alleviate postprandial hyperglycemia and have been developed into clinical anti-diabetic agents, including acarbose, miglitol, and voglibose [102]. Diarrhea and hepatotoxicity have been associated with these drugs. The low cost and relative safety of a natural source make them more suitable candidates for α-glucosidase inhibitor screening [59].

High purity CYP, 200 μg/mL, effectively increases the glucose uptake and the expression of glucose transporter two in FL838 cells also treated with tumor necrosis factor-alpha (TNF-α), restoring insulin sensitivity [103].

#### 3.2.2. Anti-Oxidative

Chinese yam autolysate and enzymatic hydrolysates (digested with pepsin, trypsin, and papain) show exceptional anti-oxidative activity [85]. Purified CYP exhibits anti-oxidative ability to scavenge hydroxyl radicals and superoxide anions, and has a reducing power of approximately one-half that of vitamin C. CYP scavenging ability increases with concentration, and purified CYP even shows anti-bacterial activity against *Escherichia coli* [104].

CYP exhibits a proliferative effect on endometrial epithelial cells in vitro, as a potential natural anti-oxidative therapeutic treatment for female infertility. Endometrial cell proliferation reaches its peak at about 36 h after 100–400 μg/mL CYP administration, due to down regulation of Bax/Bcl-2, or a pro-apoptotic/anti-apoptotic ratio [40].

Chinese yam aerial bulbil methanol extract containing batatasin I and 6,7-dihydroxy-2,4-dimethoxy phenanthrene reduced TNF-α-induced ROS production in vascular smooth muscle cells, and could be useful in preventing atherosclerotic plaque development [88]. Also, dioscorin exhibits powerful anti-oxidative activity, comparable to that of endogenous glutathione at the same concentration [83]. The anti-oxidative properties of dioscorin are greatest under acidic conditions, around pH 5 [71]. In BALB/c mice fed D-galactose to induce oxidative stress, oral administration of dioscorin was shown to attenuate oxidative stress [83].

#### 3.2.3. Anti-Inflauuatory

Chronic inflammatory pathways contribute to the pathogenesis of diabetic complications, but Chinese yam extracts have produced anti-inflammatory effects in multiple models. In pre-diabetic rats, extracts decreased levels of the pro-inflammatory cytokine IL-6 [99], while peel extract protected male Wistar rats from liver damage caused by carbon tetrachloride-induced hepatotoxicity, through its anti-inflammatory capacity [105].

The methanol extract of the Chinese yam rhizome (containing batatasin I and PAQ) exhibits anti-inflammatory activity, inhibiting both eicosanoid generation and degranulation in activated mast cells [55,89]. Both batatasin I and PAQ are dual inhibitors of COX-2 and 5-lipoxygenase (5-LOX). Dual inhibitors of “COX-2 and 5-LOX might synergistically inhibit inflammation and reduce the undesirable side effects that are associated with non-steroidal anti-inflammatory drugs” [55].

Upon oral administration of TCT to atopic dermatitis mice, atopic dermatitis was effectively suppressed through inflammatory regulation, even as the immune response was not significantly affected [90].

#### 3.2.4. Immunomodulatory

In immune regulation, CYP promotes macrophage polarization to the M1 type, thus activating their phagocytic function and secretion of proinflammatory factors [91,92]. CYP also stimulates peripheral lymphocytes, activating both CD4+ and CD8+ T cells (CD3+) for a more effective antigen-specific immune response [34,93,94].

Dioscorin exhibits immunostimulatory activity and reinforces innate immunity, stimulating nitric oxide and cytokine production, as well as enhancing phagocytosis [51]. While consumption of larger amounts can cause inflammation in vivo, smaller amounts of dioscorin may help stimulate “macrophage function and immunomodulatory effect in mucosal-associated lymphocyte tissue” [106].

#### 3.2.5. Anti-Hyperlipidemic

Hyperlipidemia is a major cause of mortality throughout the world. In addition to reducing lipid peroxidation through anti-oxidative effects, a direct effect of Chinese yam on lowering serum lipid levels has been observed. In hyperlipidemic rats fed high-fat diets with Chinese yam starch for 8 weeks, there was a significant reduction in hyperlipidemia when compared to a high-fat diet alone: serum total cholesterol decreased by 33.8%, triglycerides decreased by 46.2%, and low-density lipoprotein-cholesterol decreased by 27.5% [10]. In both rodents and humans, consuming resistant starch has a potent hypolipidemic and prebiotic effect, improving lipid profiles [95]. By weight, raw Chinese yam is more than 33% resistant starch [32].

Allantoin administration to mice fed a high-fat diet improved hepatic steatosis by activating imidazoline I1 receptors. Allantoin activation of imidazoline I1 receptors moderated hyperlipidemia in these mice by increasing LDLR gene expression and reducing ApoC-III and SREBP1c expression [96].

#### 3.2.6. Anti-Hypertensive

Intraperitoneal injections of allantoin reduced systolic blood pressure of mice fed a high-fat diet, without changing their heart rates [96]. Attributed to the activation of imidazoline I1 receptors, intravenous injections of allantoin reduced total peripheral resistance in spontaneously hypertensive rats, reducing blood pressure, heart rate, and cardiac contractility [97].

Anti-hypertensive effects of HSY aqueous extract on 2K1C renal hypertensive rats were significant, reducing mean systolic and diastolic blood pressure and preventing left ventricular hypertrophy. These benefits were attributed to a reduced angiotensin-II level, inhibited endothelin production, and activation of the in vivo antioxidant defense system [98].

#### 3.2.7. Anti-Cancer

The search for alternative medicine continues, as the “majority of chemical compounds, which have been identified as cytotoxic to cancer cells, are also toxic to normal cells” [34]. The *D. opposita* cultivar Nagaimo lectin shows anti-proliferative activity against several kinds of cancer cells when fully separated and purified. “The inhibitory activity on MCSF7 (breast cancer cells) and HepG2 (hepatoma) cells was more potent than that on CNE2 (nasopharyngeal carcinoma)” [52]. The Nagaimo lectin is especially effective against breast cancer MCSF7 cells, even provoking apoptosis. Additionally, TCT extracts showed prominent cytotoxic activities against cancer cells such as MCSF [107], A431, and HeLa cells [108].

CYMP and CYP have also been shown to have anti-cancer activity. At 10 μg/mL, CYMP treatment significantly increased mouse splenocyte-mediated cytotoxic activity against leukemia cells, and significantly increased lysosomal splenic lymphocyte production of interferon-γ. At 50 μg/mL, CYMP treatment increased lysosomal uptake capacity and phosphatase activity [34]. At 200 μg/mL, CYP significantly inhibited HepG2 and SNU-739 (both hepatoma) cell proliferation [42].

#### 3.2.8. Combination Treatments with Chinese Yam

In TCM, the Chinese yam is always used in combination with other TCM, such as Liuwei dihuang Wan, which is combined with five other TCM ingredients. Modern medicine has clinically confirmed the compatibility of 9–15 g Chinese yam, raw coix seeds, and *Poria cocos* to treat “puffiness” [109], and has also confirmed that 30 g Chinese yam and astragalus can have beneficial effects on diabetes, diarrhea, and asthma [110], that 12–15 g of Chinese yam and *Ophiopogon japonicas* can be used to treat a cough [111], and Chinese yam with apricot kernel has demonstrated clinical significance against asthma, cough, phlegm, and constipation [111]. Based on the clinical experience of modern physicians in the applications of Chinese yam, the dosage in decoctions is usually 9–50 g, while 100–200 g or more can be used for acute syndrome, severe syndrome, or rescue. Crude Chinese yam can even reach 500 g in yam congee treatment.

A Chinese yam-epidemium mixture is used to improve dyspnea, exercise ability, and the quality of life in patients with stable, moderate, or severe chronic obstructive pulmonary disease [112]. Natural and relatively inexpensive, Chinese herbal drugs are particularly suited to the long-term management of chronic diseases and should be researched further.

## 4. Discussion

The drug discovery paradox, “where breakthroughs in technology and an increasing volume of chemical and biological information have been matched by a puzzling decrease in the emergence of new drug entities”, has compelled a return to nature [113]. The main “bright spot” of innovation has involved tools used to study deoxyribonucleic acid (DNA). Previously, little was known about Chinese yam origins and phylogeny. Random amplified polymorphic DNA has been applied to understand the genetic variation of *D. opposita* cultivars [114]. Remarkably, it has been revealed that HSY is not technically *D. opposita*, but rather closer to *Dioscorea alata* L. [115]. Advantages demonstrated in genetic diversity call for “developing long-term strategies, such as modifying breeding programs through increasing utilization of wild species, conserving core germplasm, and establishing gene resource pools” [114].

The most promising of research prospects for future studies of the Chinese yam may focus on its anti-oxidative components and relate them to the control of oxidative stress, which has been implicated as a causative agent in many chronic and degenerative diseases, such as diabetes, cancer, coronary heart disease, Alzheimer’s disease, and even aging itself [29,78]. The Chinese yam has a long and storied history, both as a nutritious food source and as a source of some of the most famous TCM. Modern science is finally confirming the long-known health benefits of the Chinese yam and HSY [116]. Multi-target phytochemicals and TCM demonstrate many advantages [113]. The Chinese yam displays a myriad of distinct physiochemical properties, with exciting potential as both a food and a medicine [78].

## 5. Conclusions

This *D. opposita* review summarized its chemical compositions, bioactivities, and applications as both food and medicine. The Chinese yam presents various activities, including anti-diabetic, anti-oxidative, anti-inflammatory, immunomodulatory, anti-hyperlipidemic, anti-hypertensive, and anti-cancer functions. For thousands of years, the Chinese yam has been used to treat anorexia, chronic diarrhea, and diabetes. Therefore, the Chinese yam has been deployed in various applications as a functional food and as medicine.

## Figures and Tables

**Table 1:** Composition of Chinese yam.

Composition	Content	Description	References
Water	67−84%	-	[22]
Carbohydrates	13−27%	Mostly starch, including fiber	[10,22]
Protein	1−3%	90% Dioscorin	[23,26]
Minor molecular constituents		Abscisin II, Allantoin, Batatasins, Choline, Dopamine, Glucoprotein, Mannan, Phytic acid, Polysaccharides	[10]
Minerals		Ca, Cu, Fe, Mg, Na, P, K, Zn	[10]

**Table 2:** Nutraceutical properties of Chinese yam.

Activities	Food Type/Components	Ref.
As Food		
Anti-diabetic	Tsukuneimo	[77]
Anti-oxidative	Yams, Flour, Yogurt, Chips	[70,71,75,77,78,84]
Anti-inflammatory	Yams	[84]
Anti-hyperlipidemic	Noodles	[72]
Anti-hypertensive	Tsukuneimo	[77]
Anti-cancer	Tsukuneimo	[77]
As Medicine		
Anti-diabetic	Allantoin, Batatasin I, CYP *, TCT *	[42,43,61,86,87]
Anti-oxidative	Dioscorin, Batatasin I, CYP *	[29,40,88]
Anti-inflammatory	Batatasin I, TCT *, Cyclo-(Phe-Tyr), Cyclo-(Tyr-Tyr)	[55,69,89,90]
Immunomodulatory	CYP *, Cyclo-(Phe-Tyr), Cyclo-(Tyr-Tyr)	[69,91,92,93,94]
Anti-hyperlipidemic	Allantoin, Starch, Resistant starch	[10,95,96]
Anti-hypertensive	Allantoin, Dioscorin	[51,96,97]
Anti-cancer	Nagaimo lectin, Resistant starch, CYMP *, CYP *, Cyclo-(Phe-Tyr), Cyclo-(Tyr-Tyr)	[32,34,42,52,69]

* Abbreviations: CYP, Chinese yam; TCT, trans-*N-p*-coumaroyl tyramine; CYMP, Chinese yam mucopolysaccharide.

**Table 3:** In vivo bioactivies of Chinese yam.

Components	Bioactivities	Animals	Dosage	Duration	Ref.
Allantoin	Anti-diabetic	Streptozotocin-induced diabetic rats	0.5 mg/kg	120 min	[86]
	Anti-hyperlipidemic Anti-hypertensive	High fat diet-induced hyperlipidemic mice	Daily 5 mg/kg	4 weeks	[96]
TCT *	Anti-inflammatory	Dust mite-induced atopic dermatitis mice	Daily 50 mg/kg	4 weeks	[90]
Dioscorin	Anti-oxidantive	D-galactose-induced oxidative stress mice	Daily 20–80 mg/kg	5 Weeks	[83]
HSY * aqueous Extract	Anti-hypertensive	2K1C * renal hypertensive rats	Daily 210 mg/kg	6 weeks	[98]
Starch	Anti-hyperlipidemic	High fat diet-induced hyperlipidemic rats	Daily 6.5 g/kg	8 weeks	[10]
	Anti-diabetic	Humans	-	-	[99]
Resistant starch	Anti-hyperlipidemic	Humans	-	-	[95]

* Abbreviation: TCT, Trans-*N*-p-coumaroyl tyramine; HSY, Huai Shanyao; 2K1C, Two-kidney one-clip.

## Data Availability

No new data were created or analyzed in this study. Data sharing is not applicable to this article.

## References

[1] The Legend of the Divine Farmer. http://publicdomainreview.org/essay/the-legend-of-the-divine-farmer.

[2] Nagasawa A., Finer J.J. (1989). Plant Regeneration from Embryonic Suspension Cultures of Chinese Yam (*Dioscorea opposita* Thunb.). Plant Sci..

[3] Lee S.C., Tsai C.C., Chen J.C., Lin J.G., Hu M.L., Lu S. (2002). Effects of "Chinese yam" on hepato-nephrotoxicity of acetaminophen in rats. Acta Pharmacol. Sin..

[4] Fu Y.C., Chen S.H., Huang P.Y., Li Y.J. (2005). Application of Bubble Separation for Quantitative Analysis of Choline in *Dioscorea* (Yam) Tubers. J. Agric. Food Chem..

[5] Wang S., Yu J., Yu J., Chen H., Pang J., Liu H. (2008). Partial characterization of starches from *Dioscorea opposita* Thunb. cultivars. J. Food Eng..

[6] Ma C., Wang W., Chen Y.Y., Liu R.N., Wang R.F., Du L.J. (2005). Neuroprotective and Antioxidant Activity of Compounds from the Aerial Parts of *Dioscorea opposita*. J. Nat. Prod..

[7] Burkhill L.H. (1967). The organography and the evolution of *Dioscoreaceae*, the family of the Yams. Bot. J. Linn. Soc..

[8] The Production of Yams in the World. http://knoema.com/data/agriculture-indicators-production+yams.

[9] Chen H.L., Wang C.H., Chang C.T., Wang T.C. (2003). Effects of Taiwanese Yam (*Dioscorea japonica* Thunb. var. pseudojaponica Yamamoto) on Upper Gut Function and Lipid Metabolism in BALB/c Mice. Nutrition.

[10] Wang S., Yu J., Liu H., Chen W. (2008). Characterization and preliminary lipid-lowering evaluation of starch from Chinese yam. Food Chem..

[11] Zhang Z., Gao W., Li C., Jiang Q., Xia Y., Wang H., Huang L., Guo L. (2013). Effect of different drying methods on the physicochemical and functional properties of *Dioscorea opposita* Thunb. Starch. Starch.

[12] Luo Z., Wang Y., Jiang L., Xu X. (2015). Effect of nano-CaCO3-LDPE packaging on quality and browning of fresh-cut yam. LWT—Food Sci. Technol..

[13] Bhandari M.R., Kawabata J. (2006). Cooking effects on oxalate, phytate, trypsin and α-amylase inhibitors of wild yam tubers of Nepal. J. Food Compos. Anal..

[14] Shi K., Wu X., Ma J., Zhang J., Zhou L., Wang H., Li L. (2017). Effects of Planting and Processing Modes on the Degradation of Dithianon and Pyraclostrobin in Chinese Yam (*Dioscorea* spp.). J. Agric. Food Chem..

[15] Hsu Y.J., Weng C.F., Lin K.W., Lin K.C. (2013). Suppression of Allergic Reactions in Ovalbumin-Sensitized Mice by Yam Storage Proteins Dioscorins. J. Agric. Food Chem..

[16] Zhou Y., Zhou C., Yao H., Liu Y., Tu R. (2008). Applications of ISSR markers in detection of genetic variation among Chinese yam (*Dioscorea opposita* Thunb) cultivars. Life Sci. J..

[17] Coursey D.G. (1967). Yam Storage—I: A Review of Yam Storage Practices and of Information on Storage Losses. J. Stored Prod. Res..

[18] Ireland C.P., Schwabe W.W., Coursey D.G. (1981). The Occurrence of Batatasins in the *Dioscoreaceae*. Phytochemistry.

[19] Ozo O.N., Caygill J.C., Coursey D.G. (1984). Phenolics of Five Yam (*Dioscorea*) Species. Phytochemistry.

[20] Takasugi M., Kawashima S., Monde K., Katsui N., Masamune T., Shirata A. (1987). Antifungal Compounds from *Dioscorea batatas* Inoculated with *Pseudomonas cichorii*. Phytochemistry.

[21] Sautour M., Mitaine-Offer A.C., Miyamoto T., Wagner H., Lacaille-Dubois M.A. (2004). A New Phenanthrene Glycoside and Other Constituents from *Dioscorea opposita*. Chem. Pharm. Bull..

[22] Hashimoto T., Hasegawa K., Yamaguchi H., Saito M., Ishimoto S. (1974). Structure and Synthesis of Batatasins, Dormancy-Inducing Substances of Yam Bulbils. Phytochemistry.

[23] Zhou Y., Su Y.M., Zeng Z.H., Huang X., Zhou P. (2012). To Analyse Compatible Mechanism and Clinical Application between *Dioscorea opposita* and *Ophiopogon japonicus*. J. Basic Chin. Med..

[24] Li Y., Ji S., Xu T., Zhong Y., Xu M., Liu Y., Li M., Fan B., Wang F., Xiao J. (2023). Chinese yam (*Dioscorea*): Nutritional value, beneficial effects, and food and pharmaceutical applications. Trends Food Sci. Technol..

[25] Liu X., Bai B. (2012). Research on the Difference of Steroidal Constituents from *Dioscorea opposita* and Related Plants of the Genus *Dioscorea* L.. Henan Univ. Nat. Sci..

[26] Nagai T., Nagashima T., Suzuki N. (2007). Purification and Partial Characterization of Major Viscous Protein from Yam (*Dioscorea opposita* thunb.) Tuber Mucilage *tororo*. Int. J. Food Prop..

[27] Zhou H., Wang J., Fang X., Sun Y., Dou X. (2012). Physicochemical properties of new starches isolated from *Dioscorea opposita* Thunb. bulbils. Starch.

[28] Harvey P.J., Boulter D. (1983). Isolation and Characterization of the Storage Protein of Yam Tubers (*Dioscorea rotundata*). Phytochemistry.

[29] Hou W.C., Lee M.H., Chen H.J., Liang W.L., Han C.H., Liu Y.W., Lin Y.H. (2001). Antioxidant Activities of Dioscorin, the Storage Protein of Yam (*Dioscorea batatas* Decne.) Tuber. J. Agric. Food Chem..

[30] Jiang Q., Gao W., Li X., Xia Y., Wang H., Wu S., Huang L., Liu C., Xiao P. (2012). Characterizations of starches isolated from five different *Dioscorea* L. species. Food Hydrocoll..

[31] Zou J., Xu M., Wen L., Yang B. (2020). Structure and physiochemical properties of native starch and resistant starch in Chinese yam (*Dioscorea opposita* Thunb.). Carbohydr. Polym..

[32] Nishimura N., Tanabe H., Yamamoto T., Fukushima M. (2011). Raw Chinese Yam (*Dioscorea opposita*) Promotes Cecal Fermentation and Reduces Plasma Non-HDL Cholesterol Concentration in Rats. J. Nutr. Sci. Vitaminol..

[33] Hironaka K., Takada K., Ishibashi K. (1990). Chemical Composition of Mucilage of Chinese Yam *Dioscorea opposita* Thunb. cv. Nagaimo. Nippon Shokuhin Kogyo Gakkaishi.

[34] Choi E.M., Koo S.J., Hwang J.K. (2004). Immune cell stimulating activity of mucopolysaccharide isolated from yam (*Dioscorea batatas*). J. Ethnopharmacol..

[35] Ma F., Wang R., Li X., Kang W., Bell A.E., Zhao D., Liu X., Chen W. (2020). Physical properties of mucilage polysaccharides from *Dioscorea opposita* Thunb. Food Chem..

[36] Ma F., Wang D., Zhang Y., Li M., Qing W., Tikkanen-Kaukanen C., Liu X., Bell A.E. (2018). Characterisation of the mucilage polysaccharides from *Dioscorea opposita* Thunb. with enzymatic hydrolysis. Food Chem..

[37] Li X., Ren Z., Wang R., Liu L., Zhang J., Ma F., Khan M.Z.H., Zhao D., Liu X. (2021). Characterization and antibacterial activity of edible films based on carboxymethyl cellulose, *Dioscorea opposita* mucilage, glycerol and ZnO nanoparticles. Food Chem..

[38] Ren Z., Li X., Ma F., Zhang Y., Hu W., Khan M.Z.H., Liu X. (2022). Oil-in-water emulsions prepared using high-pressure homogenisation with *Dioscorea opposita* mucilage and food-grade polysaccharides: Guar gum, xanthan gum, and pectin. LWT—Food Sci. Technol..

[39] Ma F., Zhang Y., Wen Y., Yao Y., Zhu J., Liu X., Bell A., Tikkanen-Kaukanen C. (2017). Emulsification properties of polysaccharides from *Dioscorea opposita* Thunb. Food Chem..

[40] Ju Y., Xue Y., Huang J., Zhai Q., Wang X. (2014). Antioxidant Chinese yam polysaccharides and its pro-proliferative effect on endometrial epithelial cells. Int. J. Biol. Macromol..

[41] Zhang L., Wang M. (2017). Optimization of deep eutectic solvent-based ultrasound-assisted extraction of polysaccharides from *Dioscorea opposita* Thunb. Int. J. Biol. Macromol..

[42] Ma F., Wang R., Zhang Y., Bai J., Fang H., Ma W., Liu W., Li Q., Liu X. (2023). Polysaccharides from *Dioscorea opposita* Thunb.: Isolation, structural characterization, and anti-inflammatory and anti-tumor effects against *Hepatocellular carcinoma*. Chem. Biol. Technol. Agric..

[43] Wang R., Liu W., Liu L., Ma F., Li Q., Zhao P., Ma W., Cen J., Liu X. (2023). Characterization, in vitro digestibility, antioxidant activity and intestinal peristalsis in zebrafish of *Dioscorea opposita* polysaccharides. Int. J. Biol. Macromol..

[44] Chen X.T., Lu J., Li X., Wang Y., Miao J., Mao X.H., Zhao C.C. (2017). Effect of blanching and drying temperatures on starch-related physicochemical properties, bioactive components and antioxidant activities of yam flours. LWT—Food Sci. Technol..

[45] Lu Y.L., Chia C.Y., Liu Y.W., Hou W.C. (2012). Biological Activities and Applications of Dioscorins, the Major Tuber Storage Proteins of Yam. J. Tradit. Complement. Med..

[46] Jheng Y.J., Tsai W.Y., Chen K.H., Lin K.W., Chyan C.L., Yang C.C., Lin K.C. (2012). Recombinant dioscorins of the yam storage protein expressed in *Escherichia coli* exhibit antioxidant and immunomodulatory activities. Protein Expr. Purif..

[47] Xue Y.L., Miyakawa T., Sawano Y., Tanoura M. (2012). Cloning of genes and enzymatic characterizations of novel dioscorin isoforms from *Dioscorea japonica*. Plant Sci..

[48] Hou W.C., Liu J.S., Chen H.J., Chen T.E., Chang C.F., Lin Y.H. (1999). Dioscorin, the Major Tuber Storage Proteins of Yam (*Dioscorea batatas* Decne.), with Carbonic Anhydrase and Trypsin Inhibitor Activities. J. Agric. Food Chem..

[49] Hou W.C., Chen H.J., Lin Y.H. (2000). Dioscorins from different *Dioscorea* species all exhibit both carbonic anhydrase and trypsin inhibitor activities. Bot. Bull. Acad. Sin..

[50] Hou W.C., Chen H.J., Lin Y.H. (1999). Dioscorins, the major tuber storage proteins of yam (*Dioscorea batatas* Decne.), with dehydroascorbate reductase and monodehydroascorbate reductase activities. Plant Sci..

[51] Liu Y.W., Shang H.F., Wang C.K., Hsu F.L., Hou W.C. (2007). Immunomodulatory activity of dioscorin, the storage protein of yam (*Dioscorea alata* cv. Tainong No. 1) tuber. Food Chem. Toxicol..

[52] Chan Y.S., Ng T.B. (2013). A Lectin with Highly Potent Inhibitory Activity toward Breast Cancer Cells from Edible Tubers of *Dioscorea opposita* cv. Nagaimo. PLoS ONE.

[53] Ohizumi Y., Gaidamashvili M., Ohwada S., Matsuda K., Kominami J., Nakamura-Tsuruta S., Hirabayashi J., Naganuma T., Ogawa T., Muramoto K. (2009). Mannose-Binding Lectin from Yam (*Dioscorea batatas*) Tubers with Insecticidal Properties against *Helicoverpa armigera* (Lepidoptera: Noctuidae). J. Agric. Food Chem..

[54] Yang M.H., Chin Y.W., Yoon K.D., Kim J.W. (2013). Phenolic compounds with pancreatic lipase inhibitory activity from Korean yam (*Dioscorea opposita*). J. Enzyme Inhib. Med. Chem..

[55] Jin M.H., Lu Y., Yang J.H., Jo T.H., Park Y.I., Lee C.K., Park S.J., Son K.H., Chang H.W. (2011). Anti-inflammatory Activity of 6-Hydroxy-2,7-dimethoxy-1,4-henanthraquinone from Tuberous Roots of Yam (*Dioscorea batatas*) through Inhibition of Prostaglandin D2 and Leukotriene C4 Production in Mouse Bone Marrow-derived Mast Cells. Arch. Pharm. Res..

[56] Ramirez-Garza S.L., Laveriano-Santos E.P., Marhuenda-Munoz M., Storniolo C.E., Tresserra-Rimbau A., Vallverdu-Queralt A., Lamuela-Raventos R.M. (2018). Health Effects of Resveratrol: Results from Human Intervention Trials. Nutrients.

[57] Shankar S., Singh G., Srivastava R.K. (2007). Chemoprevention by resveratrol: Molecular mechanisms and therapeutic potential. Front. Biosci..

[58] Liao M.T., Wu C.C., Wu S.F.V., Lee M.C., Hu W.C., Tsai K.W., Yang C.H., Lu C.L., Chiu S.K., Lu K.C. (2021). Resveratrol as an Adjunctive Therapy for Excessive Oxidation Stress in Aging COVID-19 Patients. Antioxidants.

[59] Fu Y.C., Ferng L.H.A., Huang P.Y. (2006). Quantitative analysis of allantoin and allantoic acid in yam tuber, mucilage, skin and bulbil of the *Dioscorea* species. Food Chem..

[60] Mollica J.Q., Cara D.C., D’Auriol M., Oliveira V.B., Cesar I.C., Brandao M.G. (2013). Anti-inflammatory activity of American yam *Dioscorea trifida* L.f. in food allergy induced by ovalbumin in mice. J. Funct. Foods.

[61] Zhang L., Bai B., Liu X.H., Wang Y., Li M.J., Zhao D.B. (2011). α-Glucosidase inhibitors from Chinese Yam (*Dioscorea opposita* Thunb.). Food Chem..

[62] Li H., Chen K., Wang Y., Wang Y., Liu X. (2018). HPLC determination of trans-*N*-p-coumaroyltyramine in *Dioscorea opposita* Thunb. Chem. Res..

[63] Kim J.K., Heo H., Park S., Kim H., Oh J.J., Sohn E., Jung S., Lee K. (2021). Characterization of Phenethyl Cinnamide Compounds from Hemp Seed and Determination of their Melanogenesis Inhibitory Activity. ACS Omega.

[64] Rea J., Garcia-Gimenez M.D., Santiago M., De La Peurta R., Fernandez-Arche M.A. (2021). Hydroxycinnamic acid derivatives isolated from hempseed and their effects on central nervous system enzymes. Int. J. Food Sci. Nutr..

[65] Nchiozem-Ngnitedem V., Omosa L., Bedane K., Derese S., Brieger L., Strohmann C., Spiteller M. (2020). Anti-inflammatory steroidal sapogenins and a conjugated chalcon-stilbene from *Dracaena usambarensis* Engl. Fitoterapia.

[66] San H., Chaowasku T., Mekboonsonglarp W., Rodsiri R., Sritularak B., Buraphaka H., Putalun W., Likhitwitayawuid K. (2020). Constituents of *Huberantha jenkinsii* and Their Biological Activities. Molecules.

[67] Song Y.H., Kim D.W., Curtis-Long M.J., Park C., Son M., Kim J.Y., Yuk H.J., Lee K.W., Park K.H. (2016). Cinnamic acid amides from *Tribulus terrestris* displaying uncompetitive α-glucosidase inhibition. Eur. J. Med. Chem..

[68] Wang Y., Shi H., Li M., Zhao Y., Liu X. (2005). Determination of polyphenolics in *Dioscorea opposita* Thunb. and *D. alata* L.. J. Henan Univ. Nat. Sci..

[69] Belin P., Moutiez M., Lautru S. (2012). The nonribosomal synthesis of diketopiperazines in tRNA-dependent cyclodipeptide synthase pathways. Nat. Prod. Rep..

[70] Hsu C.L., Chen W., Weng Y.M., Tseng C.Y. (2003). Chemical composition, physical properties, and antioxidant activities of yam flours as affected by different drying methods. Food Chem..

[71] Chen Y.T., Kao W.T., Lin K.W. (2008). Effects of pH on the total phenolic compound, antioxidative ability and the stability of dioscorin of various yam cultivars. Food Chem..

[72] Lin J.Y., Lu S., Liou Y.L., Liou H.L. (2006). Antioxidant and hypolipidaemic effects of a novel yam-boxthorn noodle in an in vivo murine model. Food Chem..

[73] Kim S.H., Lee S.Y., Palanivel G., Kwak H.S. (2011). Effect of *Dioscorea opposita* Thunb. (yam) supplementation on physicochemical and sensory characteristics of yogurt. J. Dairy Sci..

[74] Hur S.J., Lee S.Y., Kim Y.C., Choi I., Kim G.B. (2014). Effect of fermentation on the antioxidant activity in plant-based foods. Food Chem..

[75] Lee S.Y., Ganesan P., Ahn J., Kwak H.S. (2011). *Lactobacillus acidophilus* Fermented Yam (*Dioscorea opposita* thunb.) and Its Preventative Effects on Gastric Lesion. Food Sci. Biotechnol..

[76] Nagai T., Suzuki N., Kai N., Tanoue Y. (2012). Functional properties of autolysate and enzymatic hydrolysates from yam *tsukuneimo* (*Dioscorea opposita* Thunb.) tuber mucilage *tororo*: Antioxidative activity and antihypertensive activity. J. Food Sci. Technol..

[77] Nagai T., Nagashima T. (2006). Functional Properties of Dioscorin, a Soluble Viscous Protein from Japanese Yam (*Dioscorea opposita* Thunb.) Tuber Mucilage *Tororo*. Z. Naturforsch C. J. Biosci..

[78] Chen P.Y., Tu Y.X., Wu C.T., Jong T.T., Chang C.M.J. (2004). Continuous Hot Pressurized Solvent Extraction of 1,1-Diphenyl-2-picrylhydrazyl Free Radical Scavenging Compounds from Taiwan Yams (*Dioscorea alata*). J. Agric. Food Chem..

[79] Song X., Hu H., Zhang B. (2018). Drying characteristics of Chinese Yam (*Dioscorea opposita* Thunb.) by far-infrared radiation and heat pump. J. Saudi Soc. Agric. Sci..

[80] Chang S.J., Lee Y.C., Liu S.Y., Chang T.W. (2004). Chinese Yam (*Dioscorea alata* cv. Tainung No. 2) Feeding Exhibited Antioxidative Effects in Hyperhomocysteinemia Rats. J. Agric. Food Chem..

[81] Fu Y.C., Huang P.Y., Chu C.J. (2005). Use of continuous bubble separation process for separating and recovering starch and mucilage from yam (*Dioscorea pseudojaponica* Yamamoto). LWT—Food Sci. Technol..

[82] Huang H., Jiang Q., Chen Y., Li X., Mao X., Chen X., Huang L., Gao W. (2016). Preparation, physico-chemical characterization and biological activities of two modified starches from yam (*Dioscorea opposita* Thunb.). Food Hydrocoll..

[83] Han C.H., Lin Y.F., Lin Y.S., Lee T.L., Huang W.J., Lin S.Y., Hou W.C. (2014). Effects of yam tuber protein, dioscorin, on attenuating oxidative status and learning dysfunction in D-galactose-induced BALB/c mice. Food Chem. Toxicol..

[84] Son I.S., Lee J.S., Lee J.Y., Kwon C.S. (2014). Antioxidant and Anti-inflammatory Effects of Yam (*Dioscorea batatas* Decne.) on Azoxymethane-induced Colonic Aberrant Crypt Foci in F344 Rats. Prev. Nutr. Food Sci..

[85] Nagai T., Suzuki N., Tanoue Y., Kai N., Nagashima T. (2007). Antioxidant and antihypertensive activities of autolysate and enzymatic hydrolysates from yam (*Dioscorea opposita* Thunb.) ichyoimo tubers. J. Food Agric. Environ..

[86] Niu C.S., Chen W., Wu H.T., Cheng K.C., Wen Y.J., Lin K.C., Cheng J.T. (2010). Decrease of Plasma Glucose by Allantoin, an Active Principle of Yam (*Dioscorea* spp.), in Streptozotocin-induced Diabetic Rats. J. Agric. Food Chem..

[87] Tsai C.C., Chen L.J., Niu H.S., Chung K.M., Cheng J.T., Lin K.C. (2014). Allantoin activates imidazoline I-3 receptors to enhance insulin secretion in pancreatic β-cells. Nutr. Metab..

[88] Choi K.W., Um S.H., Kwak J.H., Park H.J., Kim K.H., Moon E.Y., Kwon S.T., Pyo S.P. (2012). Suppression of adhesion molecule expression by phenanthrene-containing extract of bulbils of Chinese Yam in vascular smooth muscle cells through inhibition of MAPK, Akt, and NF-κB. Food Chem. Toxicol..

[89] Lu Y., Jin M.H., Park S.J., Son K.H., Son J.K., Chang H.W. (2011). Batatasin I, a Naturally Occurring Phenanthrene Derivative, Isolated from Tuberous Roots of *Dioscorea batatas* Suppresses Eicosanoids Generation and Degranulation in Bone Marrow Derived-Mast Cells. Biol. Pharm. Bull..

[90] Choi E.J., Ryu Y., Tang Y., Kim B., Lee W., Debnath T., Fan M., Kim E.K., Lee H.S. (2019). Effect of cinnamamides on atopic dermatitis through regulation of IL-4 in CD4+ cells. Enzyme Inhib. Med. Chem..

[91] Hao L.X., Zhao X.H. (2016). Immunomodulatory potentials of the water-soluble yam (*Dioscorea opposita* Thunb) polysaccharides for the normal and cyclophosphamide-suppressed mice. Food Agric. Immunol..

[92] Li M., Chen L.X., Chen S.R., Deng Y., Zhao J., Wang Y., Li S.P. (2017). Non-starch polysaccharide from Chinese yam activated RAW 264.7 macrophages through the Toll-like receptor 4 (TLR4)-NF-κB signaling pathway. J. Funct. Foods.

[93] Zhao G., Kan J., Li Z., Chen Z. (2005). Structural features and immunological activity of a polysaccharide from *Dioscorea opposita* Thunb roots. Carbohydr. Polym..

[94] Luo L., Qin T., Huang Y., Zheng S., Bo R., Liu Z., Xing J., Hu Y., Liu J., Wang D. (2017). Exploring the immunopotentiation of Chinese yam polysaccharide poly(Lactic-co-glycolic acid) nanoparticles in an ovalbumin vaccine formulation in vivo. Drug Deliv..

[95] Guo J., Tan L., Kong L. (2022). Multiple levels of health benefits from resistant starch. J. Agric. Food Res..

[96] Yang T.T., Chiu N.H., Chung H.H., Hsu C.T., Lee W.J., Cheng J.T. (2012). Stimulatory Effect of Allantoin on Imidazoline I1 Receptors in Animal and Cell Line. Horm. Metab. Res..

[97] Chen M.F., Tsai J.T., Chen L.J., Wu T.P., Yang J.J., Yin L.T., Yang Y.L., Chiang T.A., Lu H.L., Wu M.C. (2014). Antihypertensive Action of Allantoin in Animals. BioMed. Res. Int..

[98] Amat N., Amat R., Abdureyim S., Hoxur P., Osman Z., Mamut D., Kijjoa A. (2014). Aqueous extract of *Dioscorea opposita* Thunb. normalizes the hypertension in 2K1C hypertensive rats. BMC Complement. Altern. Med..

[99] Behall K.M., Scholfield D.J., Yuhaniak I., Canary J. (1989). Diets containing high amylose vs amylopectin starch: Effects on metabolic variables in human subjects. Am. J. Clin. Nutr..

[100] Lee H.C., Cheng W.Y., Huang B.E.G., Hsu Y.H., Huang S.Y. (2014). Anti-inflammatory and hypoglycemic efficacy of *Poria cocos* and *Dioscorea opposita* in prediabetes mellitus rats. RSC Adv..

[101] Yu J., Zhou M.N., Lu Q.J. (2015). Effects of Yam Polysaccharides on P-Selectin Expression and Macrophage Infiltration in Diabetic Nephropathy Model Rats. Clin. Exp. Med. Sci..

[102] Liu M., Yin H., Liu G., Dong J.J., Qian Z.H., Mina J.L. (2014). Xanthohumol, a Prenylated Chalcone from Beer Hops, Acts as an α-Glucosidase Inhibitor in vitro. J. Agric. Food Chem..

[103] Lee B.H., Hsu W.H., Pan T.M. (2011). Inhibitory Effects of *Dioscorea* Polysaccharide on TNF-α-induced Insulin Resistance in Mouse FL83B Cells. J. Agric. Food Chem..

[104] Yang W., Wang Y., Li X., Yu P. (2015). Purification and structural characterization of Chinese yam polysaccharide and its activities. Carbohydr. Polym..

[105] Yeh Y.H., Hsieh Y.L., Lee Y.T. (2013). Effects of Yam Peel Extract against Carbon Tetrachloride-Induced Hepatotoxicity in Rats. J. Agric. Food Chem..

[106] Lin P.L., Lin K.W., Weng C.F., Lin K.C. (2009). Yam Storage Protein Dioscorins from *Dioscorea alata* and *Dioscorea japonica* Exhibit Immunomodulatory Activities in Mice. J. Agric. Food Chem..

[107] Song Y., Mei T., Liu Y., Kong S., Zhang J., Xie M. (2021). Metabolites Identification of Chemical Constituents from the Eggplant (*Solanum melongene* L.) Calyx in Rats by UPLC/ESI/qTOF-MS Analysis and Their Cytotoxic Activities. Front. Pharmacol..

[108] Masi M., Koirala M., Delicato A., Leece D., Merindol N., Ka S., Seck M., Tuzi A., Desgagne-Penix I., Calabro V. (2021). Isolation and Biological Characterization of Homoisoflavanoids and the Alkylamide *N-p*-Coumaroyltyramine from *Crinum biflorum* Rottb., an Amaryllidaceae Species Collected in Senegal. Biomolecules.

[109] Gu C., Wang H., Piao C. (2020). Experience in the Treatment of Puffiness with Coix Seed, Poria, and Common Yam Rhizome—Three Prescription by Professor TONG, Xiaolin. Jilin J. Chin. Med..

[110] Zhou Y., Shu C.Q., Tang X., Zeng Z.H., Yang J.X. (2017). To Analysis the Significance of the Clinical Compatability Application between Shanyao and Huangqi. J. Basic Chin. Med..

[111] Zhou Y., Shu C.Q., Tang X., Zeng Z.H., Zhou P. (2017). Analysis and Clinical Compatibility Application between Shanyao and Xingren. J. Basic Chin. Med..

[112] Zhao Y.L., Song H.R., Fei J.X., Liang Y., Zhang B.H., Liu Q.P., Wang J., Hu P. (2012). The effects of Chinese Yam-Epidemium mixture on respiratory function and quality of life in patients with chronic obstructive pulmonary disease. J. Tradit. Chin. Med..

[113] Ehrman T.M., Barlow D.J., Hylands P.J. (2010). In silico search for multi-target anti-inflammatories in Chinese herbs and formulas. Bioorg. Med. Chem..

[114] Wen G.Q., Li J., Liu X.H., Zhang Y.S., Wen S.S. (2014). Extraction of total DNA and optimization of the RAPD reaction system in *Dioscorea opposita* Thunb. Genet. Mol. Res..

[115] Wu Z.G., Li X.X., Lin X.C., Jiang W., Tao Z.M., Mantri N., Fan C.Y., Bao X.Q. (2014). Genetic diversity analysis of yams (*Dioscorea* spp.) cultivated in China using ISSR and SRAP markers. Genet. Resour. Crop Evol..

[116] Li M., Li J., Liu W., Liu L., Lu J., Niu J., Liu X., Yang Q. (2014). A protocol for in vitro production of microtubers in Chinese yam (*Dioscorea opposita*). Biosci. Biotechnol. Biochem..

